# Increased Renal 2-Arachidonoylglycerol Level Is Associated with Improved Renal Function in a Mouse Model of Acute Kidney Injury

**DOI:** 10.1089/can.2016.0013

**Published:** 2016-09-01

**Authors:** Hamid Moradi, Fariba Oveisi, Elham Khanifar, Guillermo Moreno-Sanz, Nosratola D. Vaziri, Daniele Piomelli

**Affiliations:** ^1^ Division of Nephrology and Hypertension, School of Medicine, University of California, Irvine, Orange, California.; ^2^ Nephrology Section, Long Beach VA Healthcare System, Long Beach, California.; ^3^ Department of Anatomy and Neurobiology, School of Medicine, University of California, Irvine, Irvine, California.; ^4^ Long Beach Memorial Pathology Group, Long Beach, California.

**Keywords:** acute kidney injury, endocannabinoid, endocannabinoid system, inflammation, 2-arachidonoylglycerol, oxidative stress

## Abstract

**Background:** Acute kidney injury (AKI) is associated with a significantly increased risk of morbidity and mortality. Ischemia–reperfusion injury (IRI) is a major cause of AKI. In this study, we investigated the role of the endocannabinoid (EC) system in renal IRI using a well-established mouse model.

**Materials and Methods:** Renal ischemia was induced in male C57BL/6 mice by clamping both kidney pedicles for 30 min followed by 24 h of reperfusion. To increase renal 2-arachidonoylglycerol (2-AG) levels, mice were pretreated with JZL184 (16 mg/kg), 30 min before IRI. Serum creatinine and blood urea nitrogen (BUN), renal tubular damage, renal content of ECs and renal expression of markers of inflammation and oxidative stress were measured.

**Results:** Renal IRI was associated with significantly increased serum BUN and creatinine, increased tubular damage score, increased expression of renal markers of inflammation and oxidative stress and elevated renal 2-AG content. Pretreatment with JZL184 was associated with a significant increase in renal 2-AG content and there was also improved serum BUN, creatinine and tubular damage score. However, renal expression of inflammation and oxidative stress markers remained unchanged.

**Conclusions:** This is the first report documenting that renal IRI is associated with an increase in kidney 2-AG content. Further enhancement of 2-AG levels using JZL184 improved indices of renal function and histology, but did not lower renal expression of markers of inflammation and oxidative stress. Further studies are needed to determine the mechanisms responsible for the effects observed and the potential value of 2-AG as a therapeutic target in renal IRI.

## Introduction

Ischemia–reperfusion injury (IRI) is a major cause of acute kidney injury (AKI), a condition that has been reported to occur in 5–7% of hospitalized patients.^1,2^ AKI and renal IRI are associated with significantly increased risk of chronic kidney disease, delayed graft function, and mortality.^1^ Currently, there are no effective pharmacological therapies available to prevent or treat AKI, despite the major adverse health, economic, and societal impacts of this common condition.^3,4^ While multiple complex processes are involved in the pathogenesis of ischemic AKI, tubular cell injury, increased oxidant stress, and inflammation are common denominators.^5,6^ IR-induced renal tubular injury results in increased expression of adhesion molecules, such as vascular and intercellular adhesion molecule 1 (VCAM and ICAM-1), and selectins, such as P-selectin and E-selectin.^7^ This is followed by the attachment, activation, and transmigration of immune cells into renal tissue, which results in inflammation. Subsequent production of reactive oxygen species and disruption of the nitric oxide (NO) pathways lead to further tubular damage, inflammation, and oxidative stress.^7^ These abnormalities trigger a pathologic cascade of events that ultimately lead to propagation of renal injury and manifest clinically as kidney failure.

The endocannabinoids (ECs) are endogenous, bioactive lipid mediators that exert their effects mainly through specific G protein-coupled receptors: type-1 cannabinoid receptor (CB_1_) and type-2 cannabinoid receptor (CB_2_). The most extensively studied ECs are arachidonoyl ethanolamide (anandamide, AEA) and 2-arachidonoylglycerol (2-AG).^8,9^ They are synthesized on demand through distinct cellular pathways and are released in the local microenvironment, leading to autocrine or paracrine downstream effects. Given the abundance of CB_1_ receptors found in the central nervous system and CB_2_ receptors on immune cells, the ECs were initially thought to be active only in these systems. However, CB_1_ and CB_2_ receptors have been discovered in a multitude of peripheral tissue, including the kidneys.^10^ Although not fully understood, the activation of CB_1_ receptors in the periphery has been shown to be associated with increased oxidative stress and inflammation, whereas activation of CB_2_ receptor has been known to have the opposite effect.^11^ Furthermore, the ECs and their metabolites can also exert hemodynamic and other effects through CB receptor-dependent and -independent pathways.^12,13^

Given the major role that inflammation and oxidative stress play in IRI and the known involvement of the EC system in these pathways, there has been extensive evaluation of the EC system in pathophysiology of IRI of several organ systems, including the brain, heart, and the liver. Several reports indicate that both the blockade of CB_1_ receptors and the activation of CB_2_ receptors protect against IRI in the tissues mentioned.^14–17^ Interestingly, these findings have also been confirmed in nephrotoxic AKI using a murine cisplatin renal tubular injury model.^18,19^ In addition, there are now numerous reports that highlight the involvement of the EC system in renal injury and fibrosis in a variety of settings, including diabetic nephropathy.^20–23^ Despite the preponderance of evidence implicating the EC system and ECs in nonrenal IRI, data on the role of ECs in renal IRI remain sparse.^24,25^ In addition, most of the studies examining the role of the EC system in renal injury focus on the effects of the activation or inhibition of the CB receptors and do not provide data on the tissue level of the endogenous ligands for these receptors, 2-AG and AEA. In this study, we show for the first time that renal IRI leads to a significant increase in renal level of 2-AG, one of the major activators of the EC system. Furthermore, enhancement of renal 2-AG levels using pharmacologic tools improved indices of renal function without changing markers of inflammation or oxidative stress. These results indicate that renal ECs are involved in the pathogenesis of IR-induced AKI, and how modulation of the EC system may impact renal injury and function will need to be studied in further detail.

## Materials and Methods

### Chemicals

JZL184 was purchased from Cayman Chemical (Ann Arbor, MI) and dissolved in a vehicle of 4:1 PEG 300 (PEG 300: Tween 80). 2-AG was purchased from Tocris (Ellisville, MO) and further dissolved in a 1:1:1:17 ratio of DMSO:ethanol:cremophor:saline (0.9% NaCl in water). JZL184 and 2-AG were stored at −20°C. Care was taken to protect 2-AG from light. [^2^H_8_]-2-AG was purchased from Cayman Chemical. [^2^H_4_]-AEA was prepared as described previously^26^ using the appropriate fatty acid chlorides purchased from Nu-Chek Prep (Elysian, MN). AEA [ethanolamine 1-3H] was from American Radiolabeled Chemicals, Inc. (St. Louis, MO).

### Animals and experimental protocol

All animals were handled and procedures were performed in adherence to the National Institutes of Health *Guide for the Care and Use of Laboratory Animals*, and all protocols were approved by the University of California, Irvine Institutional Animal Care and Use Committee. Male C57BL/6 mice 8–12 weeks old were obtained from Jackson Laboratories (Bar Harbor, ME). They were housed in the UC Irvine facility under specific pathogen-free conditions, were allowed free access to standard chow and water, and were kept in a 12-h light:12-h dark cycle. In the first set of experiments, 20 mice were divided into two groups: one was sham operated and the other underwent 30 min of bilateral ischemia (I/R). For the second set of experiments, 20 animals were divided into two groups: one received the monoacylglycerol lipase (MGL) inhibitor JZL184 and the other received the correspondent vehicle 2 h before I/R (30 min of bilateral ischemia).

### Renal I/R injury model

We used an established mouse model of “warm” renal I/R injury.^27^ Briefly, mice were anesthetized with ketamine/xylazine (100–10 mg/kg, or to effect, i.p.) and kept on a homoeothermic station to maintain body temperature at 37°C. A warming blanket was used throughout the procedure and for 30 min postprocedure (or until the animals were awake). The body temperature of each animal was monitored closely throughout the procedure and afterward using a noninvasive infrared digital temperature device. A midline incision was made and bilateral renal pedicles were exposed. Using atraumatic Micro Serrefine straight clamps (Fine Science Tools, Inc., Foster City, CA), both renal pedicles were cross-clamped. To maintain fluid balance, mice received 0.7 mL of sterile 0.9% saline by intraperitoneal injection. After 30 min of warm ischemia, clamps were removed initiating renal reperfusion. Sham control animals were subjected to identical operation without clamping. Mice were sacrificed at 24 h after reperfusion for serum/kidney sampling under terminal general anesthesia using isoflurane. Blood was taken by intracardiac puncture after accessing the chest cavity from underneath the diaphragm. Briefly, before the opening of the chest for blood collection through cardiac puncture, anesthesia was induced in a chamber with 4–5% isoflurane in 100% O_2_ and then maintained by continuous administration of 1–2% isoflurane through nose cone. We made certain that all animals were fully anesthetized prior and during surgery. This method is approved by the University of California Irvine Institutional Animal Care and Use Committee and consistent with the AVMA Guidelines for Euthanasia. Approximately 100 μL of serum was isolated and stored at −80°C. The kidneys were harvested after euthanasia/cardiac puncture. Both kidneys were cut in half along the renal pelvis. One and half kidney was immediately snap-frozen in liquid nitrogen while half of the kidney was fixed immediately. To avoid intertissue variability and avoid comparing different regions of the kidney, we fixed the same half of the kidney (i.e., left upper half) for all animals and compared the same region of the fixed kidney for histopathology evaluation.

### Assessment of renal function and histopathology

Serum creatinine (sCr) and blood urea nitrogen (BUN) levels were measured using a kit from Bioassay Systems (Hayward, CA) following the manufacturer's protocol. For histopathology, kidney specimens were fixed in a 10% buffered formalin solution, embedded in paraffin, and sections (5 μm) were stained with hematoxylin and eosin. The histopathology scoring was performed by a trained pathologist who was unaware of experimental conditions to evaluate the degree of tubular injury based on the percentage of damaged tubules in the outer medulla, using a scale from 0 to 4: 0=<10%; 1=moderate, 10–25%; 2=severe, 25–50%; 3=very severe, 50–75%; and 4=extensive damage, >75%, as described.^27^ The tubular injury score was calculated according to the criteria: tubular dilatation, cast deposition, brush border loss, and necrosis in randomly chosen nonoverlapping fields (400×magnification).

### Lipid extraction

Frozen kidney tissues were such that equal cortex and medulla were taken from each sample, weighed and homogenized in methanol (1 mL/100 mg of tissue) containing [^2^H_8_]-2-AG and [^2^H_4_]-AEA (prepared as described previously) as internal standards. Lipids were extracted using two volumes of chloroform and one volume of water. The organic extract obtained after centrifugation at 2400 *g* for 15 min at 4°C was fractionated by open-bed silica gel column chromatography, as described.^26^ Briefly, the extract was dissolved in 2 mL of chloroform and loaded onto small glass columns packed with Silica Gel G (60-Å 230–400 Mesh ASTM; Whatman, Clifton, NJ). 2-AG and AEA were eluted with 2 mL of chloroform/methanol (9:1, v/v). The lipids were collected and dried under nitrogen, and the lipid pellet was reconstituted in 60 μL of methanol.

### Liquid chromatography/mass spectrometry

Tissue levels of 2-AG and AEA were quantified by LC/MS.^26^ An 1100-LC system coupled with a 1946A-MS detector (Agilent Technologies, Inc., Palo Alto, CA) equipped with an electrospray ionization interface was used. 2-AG and AEA were eluted on a XDB Eclipse C18 column (50×4.6 mm inner diameter, 1.8 μm, Zorbax; Agilent Technologies, Inc.) using a linear gradient of methanol in water (from 85% to 90% methanol in 2.5 min), at a flow rate of 1 mL/min. Column temperature was kept at 40°C. MS detection was in the positive ionization mode, capillary voltage was set at 3000 V, and fragmentor voltage varied from 120 to 140 V. Nitrogen was used as drying gas at a flow rate of 13 L/min and a temperature of 350°C. Nebulizer pressure was set at 60 psig. For quantification purposes, we monitored, in the selective ion-monitoring mode, the Na^+^ adducts ([M + Na^+^]) of [^2^H_8_]-2-AG (mass-to-charge ratio, *m/z*, 409), 2-AG (*m/z*, 401), [^2^H_4_]-AEA (*m/z*, 374), and AEA (*m/z*, 370).

### RNA extraction and real-time polymerase chain reaction

Total RNA extraction was carried out using the upper half of the kidney making sure that the same amount of cortex and medulla was taken from each sample. TRIzol reagent (Life Technologies, Carlsbad, CA) was used according to the manufacturer's instructions. Genomic DNA was subsequently removed using DNAse I following the manufacturer's protocol (Life Technologies). Reverse transcription and real-time polymerase chain reaction (PCR) were performed with 0.2 μg of DNA-free RNA and the iTaq™ Universal SYBR^®^ Green One-Step Kit (Bio-Rad). Transcript levels of genes were measured by real-time PCR (fluorescence detection of SYBR green) in an iCycler iQ apparatus (Bio-Rad). mRNA levels were normalized using GAPDH as an endogenous control. The primer sequences of the analyzed genes are listed in Table 1.

**Table 1.** **Primers Used for RT-PCR Analysis**

Gene name	Forward Primer (5′-3′)	Reverse Primer (5′-3′)
NADPH oxidase 2	TGCCAGTCTGTCGAAATCTGC	ACTCGGGCATTCACACACC
NADPH oxidase 4	CCTTTTACCTATGTGCCGGAC	CATGTGATGTGTAGAGTCTTGCT
GPx1	GTGCAATCAGTTCGGACACCA	CACCAGGTCGGACGTACTTG
SOD1	TATGGGGACAATACACAAGGCT	CGGGCCACCATGTTTCTTAGA
SOD2	TGGACAAACCTGAGCCCTAAG	CCCAAAGTCACGCTTGATAGC
SOD3	GATGGAGCTAGGACGACGAAG	CAGCGTGGCTGATGGTTGTA
CAT	GGAGGCGGGAACCCAATAG	GTGTGCCATCTCGTCAGTGAA
ICAM	TGCCTCTGAAGCTCGGATATAC	TCTGTCGAACTCCTCAGTCAC
IL-8	TGTTGAGCATGAAAAGCCTCTAT	AGGTCTCCCGAATTGGAAAGG
IL-1beta	GAAATGCCACCTTTTGACAGTG	TGGATGCTCTCATCAGGACAG
TNF-alpha	CAGGCGGTGCCTATGTCTC	CGATCACCCCGAAGTTCAGTAG
IL-6	TCTATACCACTTCACAAGTCGGA	GAATTGCCATTGCACAACTCTTT
VCAM	TTCGGTTGTTCTGACGTGTG	TACCACCCCATTGAGGGGAC
P-selectin	CATCTGGTTCAGTGCTTTGATCT	ACCCGTGAGTTATTCCATGAGT
E-selectin	CTCGGGCATGTGGAATGAC	TGCATTGGTACACGAAGCTGT
GAPDH	TGACCTCAACTACATGGTCTACA	CTTCCCATTCTCGGCCTTG

NADPH oxidase, nicotinamide adenine dinucleotide phosphate oxidase; Gpx1, glutathione peroxidase 1; SOD, superoxide dismutase; CAT, Catalase; IL, interleukin; ICAM, intercellular adhesion molecule; VCAM, vascular cell adhesion molecule; TNF, tumor necrosis factor; P-selectin, platelet-selectin; e-selectin, endothelial-selectin; GAPDH, glyceraldehyde 3-phosphate dehydrogenase.

### Data analyses

Statistical analyses were done using GraphPad Prism software (GraphPad Software, San Diego, CA). Student's *t*-test was used in statistical evaluation of the data, which are shown as mean ± standard error of the mean. *p*-values <0.05 were considered significant. In regard to correlation analyses, we performed two statistical tests, the D'Agostino Pearson Omnibus normality test and Shapiro–Wilk normality test to determine if BUN and creatinine are normally distributed. Once we confirmed that both of these indices passed the normality test, we then used a Pearson correlation coefficient to determine the strength of the linear relationship between these two variables and 2-AG. For renal histology, we used one way ANOVA Kruskal–Wallis test to determine the statistical significance of our findings (***p<0.001).

## Results

### General data

**FIG. 1.**
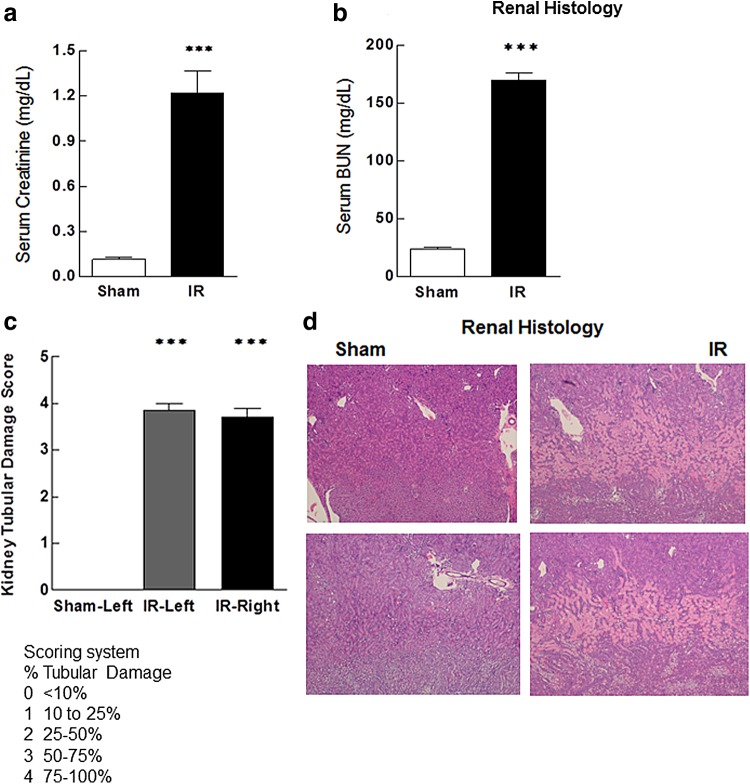
Serum markers of renal function, BUN, and creatinine are significantly elevated in animals with IRI compared with sham-operated controls **(a, b)**. Renal histology shows significant tubular damage at the renal corticomedullary junction in animals with IRI when compared with sham-operated controls **(c, d)**. Damage was evaluated and expressed in the following scoring system: scoring system % damage; 0, <10%; 1, 10–25%; 2, 25–50%; 3, 50–75%; 4, 75–100%. Data are expressed as mean ± SEM, *n*=6–10 animals in each group ***p<0.005. BUN, blood urea nitrogen; IRI, ischemia–reperfusion injury; SEM, standard error of the mean.

As expected, renal IRI resulted in severe renal dysfunction marked by significant elevation of sCr and BUN levels in animals undergoing IRI, when compared with sham-operated controls (Fig. 1). In addition, IRI was associated with severe renal tubular damage as evidenced by widespread acute tubular necrosis, loss of the brush border, cast formation, and tubular dilation at the corticomedullary junction (Fig. 1).

### Impact of renal IRI on renal inflammation and oxidative stress

**FIG. 2.**
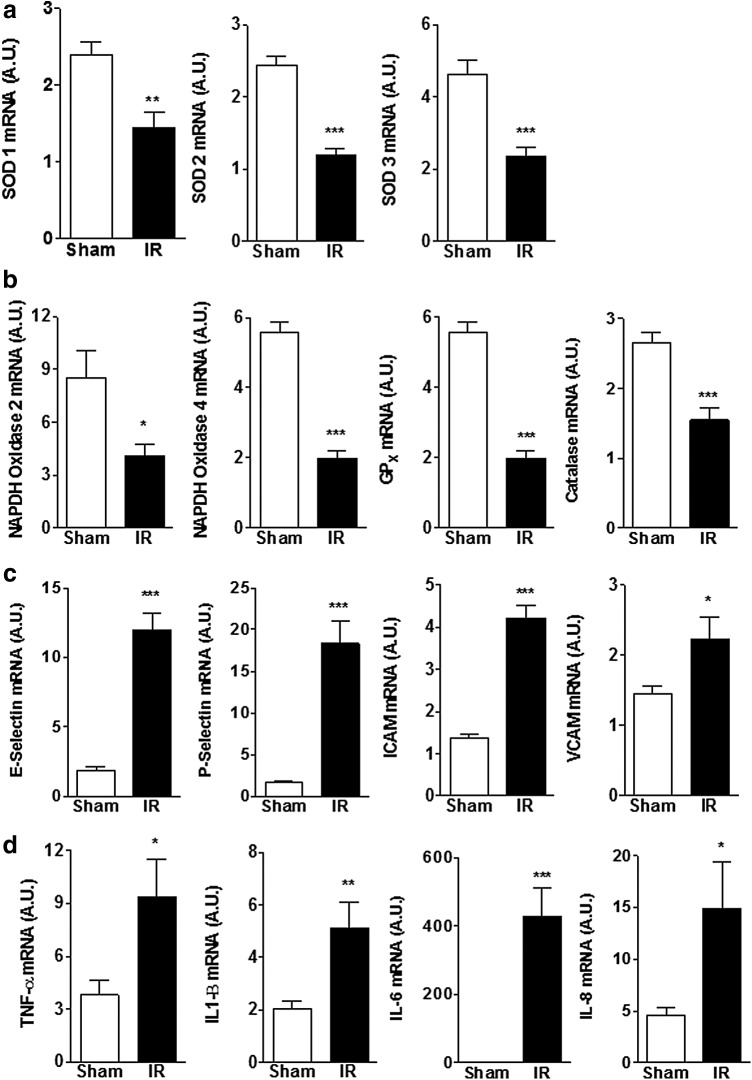
Renal mRNA expression of proteins involved in the oxidative stress pathway **(a, b)** and inflammation **(c, d)** in animals with IRI compared to sham-operated controls. Expression of antioxidant enzymes decreased, while expression of proinflammatory cytokines, adhesion molecules, and pro-oxidative stress enzymes increased in animals with IRI. Data are expressed as mean ± SEM, *n*=6–10 animals in each group *p<0.05, **p<0.01, ***p<0.005.

Consistent with the available literature, animals with renal IRI had a significant increase in renal expression of pro-oxidant enzymes (NADPH oxidase 2 and 4), whereas renal expression of antioxidant enzymes was significantly reduced relative to sham-operated controls (Fig. 2a, b). Furthermore, IRI was associated with a significant increase in the expression of adhesion molecules and markers of inflammation such as inflammatory cytokines (Fig. 2c, d).

### Impact of renal IRI on tissue EC levels

**FIG. 3.**
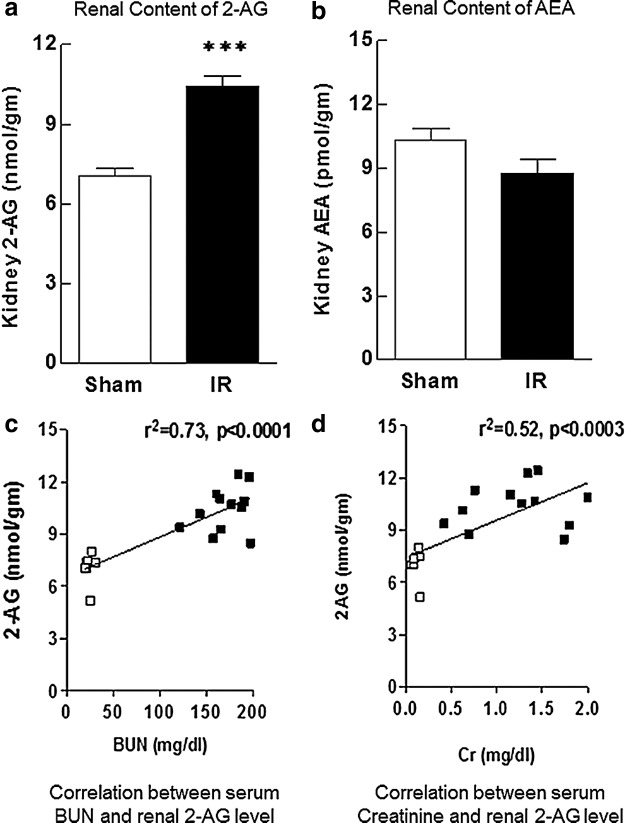
Renal content of 2-AG increased significantly 24 h after IRI, while AEA content did not change in animals post-IRI compared with sham-operated controls **(a, b)**. Serum BUN and creatinine strongly and significantly correlated with renal 2-AG content in both groups **(c, d)**. Data are expressed as mean±SEM, *n*=6–10 animals in each group ***p<0.005, *R*^2^>0.5 was considered significant. 2-AG, 2-arachidonoylglycerol.

Animals undergoing renal IRI had significantly increased kidney 2-AG content when compared with sham-operated controls (Fig. 3). There was also a significant correlation between renal 2-AG levels and markers of renal function such as BUN and creatinine. By contrast, renal AEA content marginally decreased with IRI, but this change did not reach statistical significance.

### Impact of JZL184 treatment on renal 2-AG level and renal injury

**FIG. 4.**
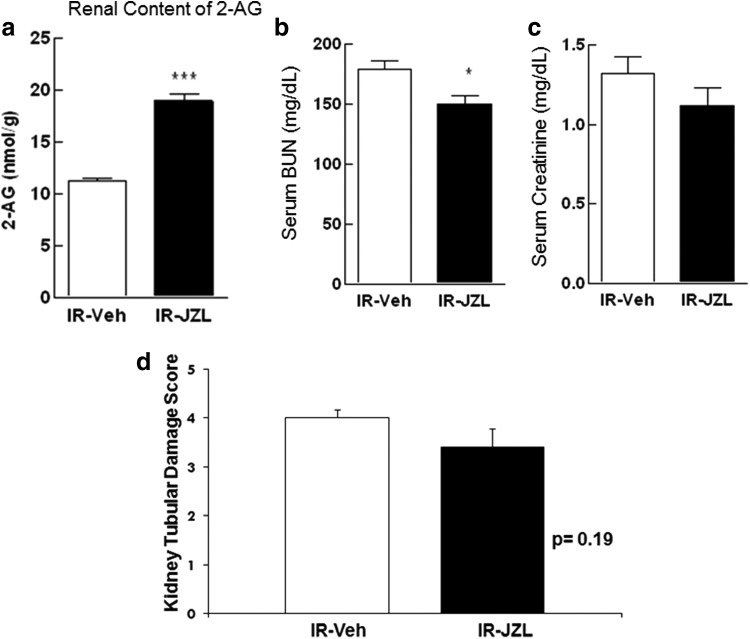
Renal 2-AG content increased significantly 24 h post-IRI in animals treated with JZL184 (2 h before induction of IR) compared to vehicle-treated controls who also underwent IRI **(a)**. Plasma markers of renal function (BUN and creatinine) and renal histology (tubular damage score) improved in animals treated with JZL184 **(b–d)**. Data are expressed as mean ± SEM, *n*=6–10 animals in each group, *p<0.05, ***p<0.005.

Given the significant increase in renal 2-AG levels in animals with IRI and to examine the potential role of 2-AG in renal dysfunction and damage of ischemic AKI, a different group of animals was treated with the compound JZL184, a highly potent inhibitor of MGL, the 2-AG-hydrolyzing enzyme. Treatment with JZL184 has been previously shown to increase 2-AG levels in several mouse tissues.^28^ Animals were treated with JZL184 (16 mg/kg) or vehicle 2 h before ischemic injury and compared after 24 h. As expected, treatment with JZL184 was associated with a significant increase in renal 2-AG content when compared with vehicle-treated controls 24 h post-IRI (Fig. 4). Pretreatment of animals with JZL184 was also associated with a significant improvement in serum BUN concentrations. There was also a modest improvement in sCr and renal histology in animals treated with JZL184 when compared with vehicle-treated controls (Fig. 4).

**FIG. 5.**
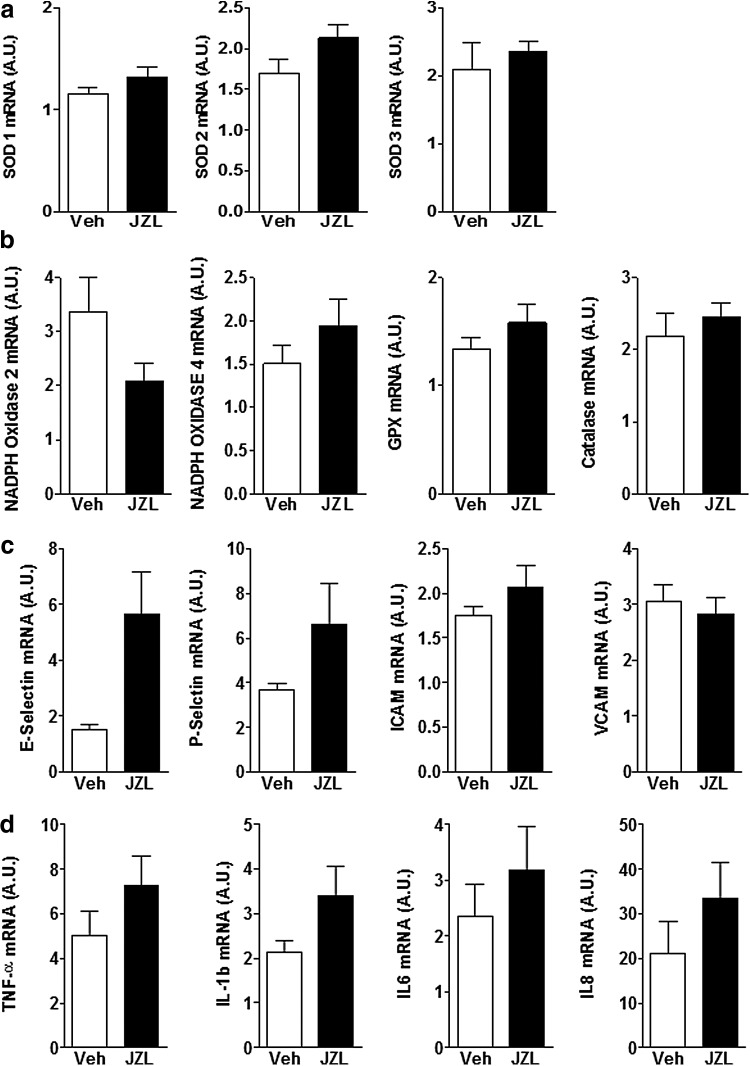
Renal expression of markers of oxidative stress pathway **(a, b)** and inflammation **(c, d)** did not change 24 h post-IRI in animals treated with JZL184 2 h before induction of IR compared to vehicle-treated controls. Data are expressed as mean±SEM, *n*=6–10 animals in each group.

Next, we examined the expression of renal adhesion molecules, inflammatory cytokines, and antioxidant enzymes to determine whether 2-AG-mediated improvement in renal function and histology might be facilitated through alleviation of renal inflammation and oxidative stress. We found that enhanced renal 2-AG levels did not change expression of markers of renal inflammation and oxidative stress, and there was a trend toward worsening of inflammation, although this did not reach statistical significance (Fig. 5). In addition, renal expression of antioxidant pathways was mainly unchanged in mice treated with JZL184 when compared with controls.

## Discussion

This is the first study that examines the effect of kidney IRI on renal EC levels and their potential impact on pathophysiology of AKI. We have found that kidney IRI is associated with a significant increase in renal 2-AG content. Kidney 2-AG levels correlated positively and significantly with serum BUN and creatinine. Furthermore, enhancement of renal 2-AG levels using the selective MGL inhibitor, JZL184, caused a modest but significant improvement in renal function. Interestingly, the latter findings were not associated with improvement in renal markers of inflammation and oxidative stress, indicating that the improvement in renal function induced by 2-AG may be independent of proinflammatory and oxidative processes.

The findings of our study are unique in several respects. First, while the role of the EC system in AKI has been examined through modulation of CB receptors in animal models of nephrotoxic tubular injury, this is the first investigation examining EC levels and the role of the EC system in ischemic AKI and renal IRI.^18,19,24^ Therefore, the current findings shed light on how the EC system as a whole may be involved in the changes observed in renal IRI. Furthermore, our study demonstrates that renal IRI is associated with increased kidney 2-AG content. The latter findings are significant because understanding how ECs are mobilized in renal IRI is crucial to their potential exploitation as a therapeutic strategy. It is also intriguing to note that increased tissue 2-AG content has also been reported in IRI in other organ systems such as the liver, and, just as in the kidney, enhancement of tissue 2-AG levels was associated with improvement of IRI.^17,29–31^ Therefore, it is noteworthy that the findings described in this study are not exclusive to the kidney, thus reflecting a potential physiopathological role of 2-AG and the EC system in IRI, which needs to be further explored.

While increased 2-AG levels have mostly been reported to be associated with reduced tissue injury in IR, the mechanisms through which 2-AG affords protection in IRI remain unclear. Several possibilities have been examined, one of them being a potential anti-inflammatory action of 2-AG mediated by CB_2_ receptors. However, available data on the impact of 2-AG on inflammation are contradictory, with some studies reporting anti-inflammatory properties, while others noting proinflammatory effects.^32–36^ In our study, we did not find significant changes in the expression of proinflammatory cytokines or adhesion molecules following pharmacological enhancement of renal 2-AG levels. In fact, we observed a trend toward an increased expression of these markers. Indeed, there are recent studies that link increased 2-AG levels with worsening of inflammation.^37,38^ It is possible that increased tissue levels of 2-AG may lead to activation of CB_1_ receptor, hence causing a trend toward worsening inflammation; however, this mechanism has not been established in renal IRI. Given that indices of renal inflammation and oxidative stress remain unchanged, our findings support the notion that the modest improvement in renal function observed with enhanced 2-AG levels may be related to effects independent of its role in inflammation and oxidative stress. In this regard, there are several studies indicating that 2-AG has vasodilatory properties through CB_1_-dependent and -independent mechanisms.^13,39^ For instance, Awumey et al. have demonstrated that 2-AG can cause relaxation of arterial smooth muscle through its metabolite glycerated epoxyeicosatrienoic acid, which can activate potassium-gated calcium channels on vascular smooth muscle cells resulting in hyperpolarization of these cells and vascular relaxation.^40^ One possibility is that increased kidney 2-AG levels in renal IRI could cause arterial vasodilatation, which would lead to improved renal perfusion and enhanced glomerular filtration rate, thereby explaining the improvement observed in renal function. It should also be noted that since we administered JZL184 systemically and most likely increased 2-AG levels in other parts of the body, it is possible that the renoprotective effect observed in our study may be emanating from outside of the kidneys (i.e., modulation of sympathetic nervous system output). These possibilities will need to be examined in future studies.

The study of the EC system in renal disease is important for several reasons. First, there is emerging evidence that dysregulation of this system may be involved in diabetic nephropathy, proteinuria, renal fibrosis, and AKI.^20–23^ Second, pharmacological agents are available, which can modulate EC levels and CB receptor activity, thereby providing potential therapeutic strategies. Finally, considering recent reports pointing at synthetic cannabinoid use as a cause of AKI,^41^ investigation of the EC system in renal disease may shed light on the mechanism by which these recreational drugs can potentially cause renal injury and help formulate preventive plans in risk population.

Several limitations of our study need to be mentioned. The potential role of 2-AG as a mediator of vasodilation and its impact on renal blood flow rate will need to be thoroughly explored in future studies. Furthermore, in our mouse model of AKI, we had a limited supply of plasma, and therefore, systemic levels of ECs in IR AKI remain to be determined. Moreover, given that JZL184 was administered systemically, we cannot rule out inhibition of MGL in other organs that could have had a downstream effect on the kidneys. In addition, despite its specificity for MGL, it is possible that JZL184 may have an impact on other serine hydrolase enzymes that may explain some of the results we are observing in our studies. Furthermore, our evaluation of renal markers of inflammation and oxidative stress pathways was limited to mRNA analysis, and therefore, renal abundance of each protein will need to be determined to complement the mRNA findings. Finally, our study does not address the mechanism/s responsible for increased renal 2-AG levels. We have recently reported that oxidative stress can cause the reversible sulfenylation of MGL and inhibition of its activity, hence leading to decreased 2-AG breakdown and increased 2-AG levels.^42^ Given that kidney injury (whether acute or chronic) is associated with significant oxidative stress, it is possible that MGL inhibition is the mechanism responsible for increased 2-AG levels in renal IRI, however, this possibility will need to be confirmed in future studies.

In conclusion, renal IRI is associated with a significant increase in kidney 2-AG content. Further enhancement of renal 2-AG levels using a pharmacologic tool, which inhibits its breakdown, improves indices of renal function and kidney injury, without affecting expression of markers of inflammation and oxidative stress. Future studies will need to elucidate the mechanisms responsible for increased renal 2-AG levels in IRI and its potential utility as a therapeutic agent.

## Abbreviations Used

2-AG: 2-arachidonoylglycerol
AKI: acute kidney injury
BUN: blood urea nitrogen
EC: endocannabinoid
ICAM-1: intercellular adhesion molecule 1
IRI: ischemia–reperfusion injury
LC/MS: liquid chromatography/mass spectrometry
MGL: monoacylglycerol lipase
NO: nitric oxide
PCR: polymerase chain reaction
sCr: serum creatinine
SEM: standard error of the mean
VCAM: vascular cell adhesion molecule
